# Breath-by-breath P0.1 measured on quasi-occlusion via Hamilton C6 may result in underestimation of respiratory drive and inspiratory effort

**DOI:** 10.1186/s13054-022-04286-5

**Published:** 2022-12-26

**Authors:** Ryo Takane, Mikio Nakajima, Maki Miwa, Richard H. Kaszynski, Tomotsugu Nakano, Hideaki Goto, Muneyuki Takeuchi

**Affiliations:** 1 Emergency and Critical Care Center, Tokyo Metropolitan Hiroo Hospital, 2−34−10, Ebisu, Shibuya-Ku, Tokyo, 150-0013 Japan; 2 Department of Intensive Care Medicine, Osaka Women’s and Children’s Hospital, Osaka, Japan

**Keywords:** P0.1, Esophageal pressure, Occlusion pressure, Swing, Respiratory drive, Inspiratory effort, COVID-19, Mechanical ventilation

## Abstract

We aimed to identify the threshold for P0.1 in a breath-by-breath manner measured by the Hamilton C6 on quasi-occlusion for high respiratory drive and inspiratory effort. In this prospective observational study, we analyzed the relationships between airway P0.1 on quasi-occlusion and esophageal pressure (esophageal P0.1 and esophageal pressure swing). We also conducted a linear regression analysis and derived the threshold of airway P0.1 on quasi-occlusion for high respiratory drive and inspiratory effort. We found that airway P0.1 measured on quasi-occlusion had a strong positive correlation with esophageal P0.1 measured on quasi-occlusion and esophageal pressure swing, respectively. Additionally, the P0.1 threshold for high respiratory drive and inspiratory effort were calculated at approximately 1.0 cmH_2_O from the regression equations. Our calculations suggest a lower threshold of airway P0.1 measured by the Hamilton C6 on quasi-occlusion than that which has been previously reported.

## Introduction

During invasive mechanical ventilation, high respiratory drive may be associated with strong inspiratory effort and negative pleural pressure (high transpulmonary pressure), which may cause patient self-inflicted lung injury (P-SILI) [1]. Although the most accurate method for assessing respiratory drive and inspiratory effort requires esophageal pressure measurement, the method comes with certain drawbacks such as resource- and labor-intensiveness.

P0.1 measures the airway pressure 100 ms after the initiation of an inspiratory effort during an airway occlusion and is frequently adopted as a surrogate index for respiratory drive. This measurement utilizes the coincidence of changes in intrathoracic and intra-airway pressure due to airway occlusion. P0.1 measured with airway occlusion correlates with inspiratory effort and respiratory drive [2], but because the degree of spontaneous effort changes breath-by-breath, this method is limited to the sampled breath involving airway occlusion. In response to this inherent limitation, Hamilton ventilators come equipped with a function to measure breath-by-breath P0.1 based on the short quasi-occlusion associated with inspiratory triggering [3]. However, measurements achieved without complete airway occlusion may result in systematic underestimation of actual P0.1 [2], which may involve a lower threshold for defining high respiratory drive. The appropriate threshold for P0.1 measured on quasi-occlusion for high respiratory drive has yet to be defined. Therefore, the aim of the present study is to identify the threshold for high respiratory drive that is valid for P0.1 measured on the short quasi-occlusion period by Hamilton C6 associated with inspiratory triggering.

## Methods

This is a single-center prospective observational study conducted from March to September 2021. Eligible patients were as follows: (i) adults (aged ≥ 18), (ii) diagnosed with COVID-19 with a positive real-time polymerase chain reaction for SARS-CoV-2, (iii) receiving invasive mechanical ventilation with patient’s spontaneous breathing and (iv) underwent insertion of an esophageal balloon catheter. We measured airway P0.1 on quasi-occlusion (P0.1aw), esophageal P0.1 (P0.1es) and esophageal pressure swing (ΔPes). These measurements were repeated multiple times for each patient and performed via Hamilton C6 ventilators (Hamilton Medical AG, Bonaduz, Switzerland) and NutriVent multifunction naso-gastric catheters (Sidam, Mirandola, Italy). Reliability of the esophageal pressure measurements was confirmed using the dynamic occlusion test. The acceptable range of the ∆Pes/∆Paw ratio during occlusion test was defined as 0.8–1.2 [4]. For each patient, P0.1aw, P0.1es and ΔPes were documented when level of sedation or ventilator settings were changed. The Hamilton C6 automatically calculates breath-by-breath P0.1aw and P0.1es from the maximum slope of the Paw and Pes drops taking place during the quasi-occlusion interval associated with inspiratory triggering [3]. We recorded the average P0.1aw and P0.1es values of five consecutive breaths displayed on the ventilator. ΔPes was manually measured from the graph displayed on the ventilator and recorded as the average of five consecutive breaths.

We devised scatter plots between (i) P0.1aw and P0.1es, and (ii) P0.1aw and ∆Pes. We analyzed whether there was a linear relationship between the two variables. Repeated measures correlation was performed because multiple measurements were taken on individual patients [5]. A *P*-value of less than 0.05 was considered statistically significant. Then, we conducted a linear regression analysis and derived the threshold of P0.1aw for high respiratory drive and inspiratory effort based on this regression model. To account for clustering within patients, a generalized linear model was linked to the analysis (generalized estimating equation, GEE). High respiratory drive was defined as two distinct thresholds: P0.1es 3.5 and 4.0 cmH_2_O [6]. High inspiratory effort was also defined by two distinct thresholds: ∆Pes 10 and 15 cmH_2_O [7, 8]. Additionally, a Bland–Altman plot visualizing the differences between P0.1aw and P0.1es versus their average was constructed to evaluate agreement between these two values [8]. Analyses were conducted using R version 4.1.2 and Stata BE/17.

## Results

**Fig. 1:**
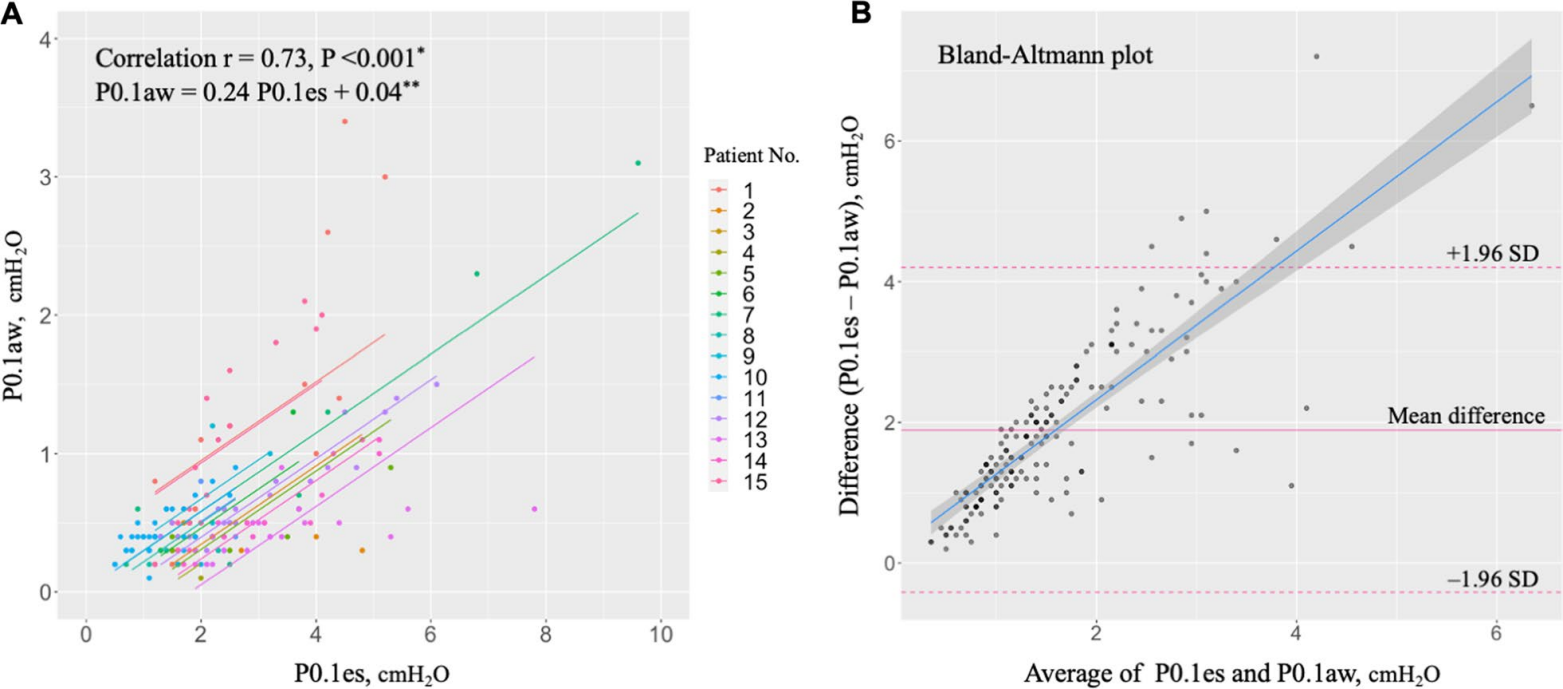
Relationship between airway P0.1 and esophageal P0.1 measured on quasi-occlusion. **A** Correlation between airway P0.1 (P0.1aw) and esophageal P0.1 (P0.1es). Each value was measured breath-by-breath on quasi-occlusion via Hamilton C6 ventilator. *Repeated measures correlation. **Generalized linear model accounting for clustering within patients. **B** Bland–Altmann plot of differences between P0.1aw and P0.1es versus their average. SD, standard deviation of mean difference (15 patients, 172 measurements)

We included 15 patients and collected 172 measurements. The characteristics of the patients are shown in Table 1. The median age of patients was 58 years and in-hospital mortality was 40%. Figure 1A shows the relationship between P0.1aw and P0.1es with parallel lines fitted for each patient. P0.1aw was significantly correlated with P0.1es (*r* = 0.73, *P* < 0.001). Regression analysis (GEE) revealed that P0.1aw was equivalent to 0.24-fold of P0.1es (P0.1aw = 0.24 P0.1es + 0.04). The P0.1aw thresholds for high respiratory drive were calculated as 0.88 and 1.00 cmH_2_O from the regression equations. These two values were found for the two different P0.1es thresholds selected to define high respiratory drive of P0.1es 3.5 and 4.0 cmH_2_O, respectively. Figure 1B shows a Bland–Altmann plot illustrating the differences between P0.1aw and P0.1es versus their average. The blue line indicates a fitted liner regression model with standard error between the difference and average. The Bland–Altman plot revealed a proportional error between the two values. The proportional error can be observed on the Bland–Altmann plot as a widening trend in the differences between P0.1aw and P0.1es versus their average.

**Table 1:** Patient characteristics (*N* = 15)

Variables	Median *n*	[IQR] (%)
Age, years	58	[43–73]
Male	11	(73%)
Body mass index	27.8	[24.9–33.3]
Duration from onset of COVID-19 symptoms to intubation, days	9	[7–13]
Past medical history		
Diabetes mellitus	7	(47%)
Hypertension	5	(33%)
Dyslipidemia	5	(33%)
Cardiovascular disease	3	(20%)
Chronic obstructive pulmonary disease	1	(7%)
Smoking	5	(36%)
Ventilator setting, cmH_2_O		
PEEP	13	[10–15]
Pressure control/support (above PEEP)	12	[10–15]
Peak inspiratory pressure	23	[22–30]
PaO_2_/F_I_O_2_ ratio	180	[156–256]
Extracorporeal membrane oxygenation	1	(7%)
In-hospital death	6	(40%)

Data are shown as *n* (%) or median [IQR]

*PEEP* positive end-expiratory pressure, *IQR* interquartile range

**Fig. 2:**
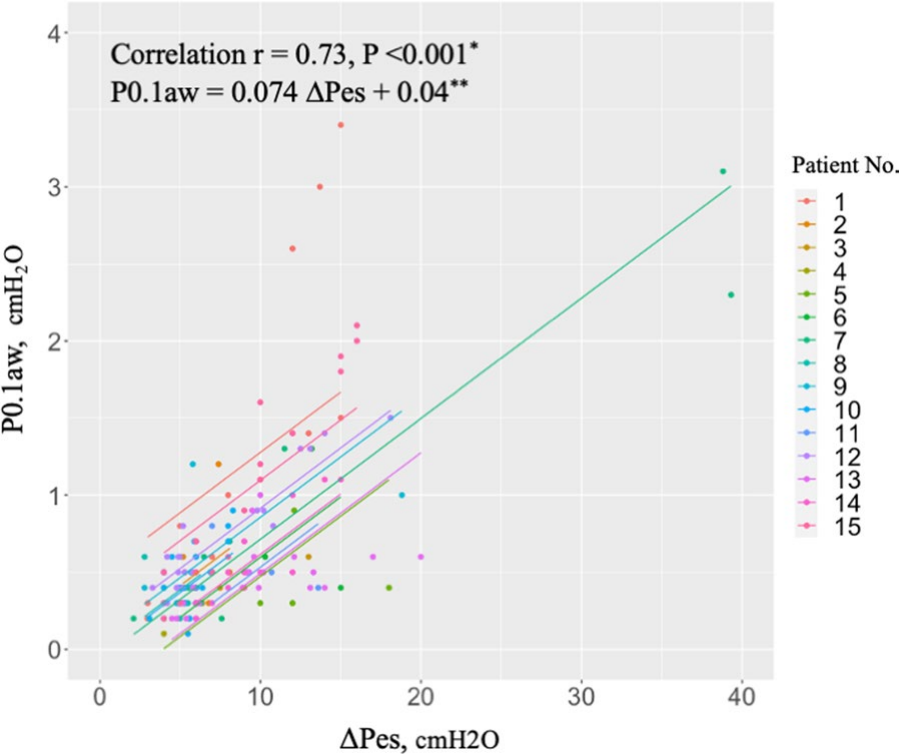
Relationship between airway P0.1 measured on quasi-occlusion and esophageal pressure swing. Correlation between airway P0.1 (P0.1aw) and esophageal pressure swing (∆Pes). P0.1aw was measured breath-by-breath on quasi-occlusion via Hamilton C6 ventilator. *Repeated measures correlation. **Generalized linear model accounting for clustering within patients (15 patients, 172 measurements)

The relationship between P0.1aw and ∆Pes is shown in Fig. 2. Parallel lines are fitted for each patient. There was a positive correlation between P0.1aw and ∆Pes, which was statistically significant (*r* = 0.73, *P* < 0.001). The P0.1aw thresholds for high inspiratory effort were calculated as 0.78 and 1.15 cmH_2_O using GEE (P0.1aw = 0.074 ∆Pes + 0.04). These two values were found for the two different ∆Pes thresholds selected to define high inspiratory effort of ∆Pes 10 and 15 cmH_2_O, respectively.

## Discussion

In the present study, we found that breath-by-breath P0.1aw measured by the Hamilton C6 on quasi-occlusion significantly correlated with P0.1es and ∆Pes. The threshold value of P0.1aw for high respiratory drive was calculated at approximately 1.0 cmH_2_O from both regression equations. Additionally, P0.1aw was equivalent to a quarter of P0.1es.

A previous study reported the nominal range for P0.1aw measured with airway occlusion to be between 0.5 and 1.5 cmH_2_O [9]. Another previous study reported airway P0.1 with occlusion above 3.5 cmH_2_O to be associated with high respiratory drive [10]. In contrast, the airway P0.1 measured on quasi-occlusion may result in underestimation of these values [2]. Our findings suggest a lower threshold of P0.1 measured on quasi-occlusion utilizing the Hamilton C6 than that which has been previously reported.

It should be noted that clinical threshold of the P0.1 value for high respiratory drive and inspiratory effort described in the present study is not universally applicable to other ventilators. This is because we exclusively assessed P0.1 measurements using a single ventilator (Hamilton C6). The generalizability would be limited since each manufacturer utilizes its own proprietary software to estimate P0.1. For instance, Getinge group Servo I and U ventilators also provide P0.1 values without airway occlusions. Beloncle et al. [11] showed that the absolute values of P0.1 measurements vary between ventilator manufacturers. It should be further highlighted that under ideal circumstances as of November 2022, airway P0.1 with occlusion should be adopted as a standard reference; however, the Hamilton C6 ventilator does not include a function to measure airway P0.1 with occlusion. Therefore, we adopted esophageal P0.1 and esophageal swing for high respiratory drive and inspiratory effort, respectively as surrogates [6].

In patients with COVID-19, the evaluation of respiratory drive and inspiratory effort are critical due to concerns of P-SILI. A P0.1aw value measured on quasi-occlusion of approximately 1.0 cmH_2_O or higher may suggest a high respiratory drive and inspiratory effort. P0.1aw may be equivalent to a quarter of P0.1es on Hamilton C6.

## Data Availability

Not available.

## Abbreviations

P-SILI: Patient self-inflicted lung injury
P0.1aw: Airway P0.1 measured on quasi-occlusion
P0.1es: Esophageal P0.1 measured on quasi-occlusion
∆Pes: Esophageal pressure swing
